# Sex-Biased Gene Expression during Head Development in a Sexually Dimorphic Stalk-Eyed Fly

**DOI:** 10.1371/journal.pone.0059826

**Published:** 2013-03-19

**Authors:** Gerald S. Wilkinson, Philip M. Johns, Jackie D. Metheny, Richard H. Baker

**Affiliations:** 1 Department of Biology, University of Maryland, College Park, Maryland, United States of America; 2 Biology Program, Bard College, Annandale-on-Hudson, New York, United States of America; 3 Sackler Institute for Comparative Genomics, American Museum of Natural History, New York, New York, United States of America; University of Arkansas, United States of America

## Abstract

Stalk-eyed flies (family Diopsidae) are a model system for studying sexual selection due to the elongated and sexually dimorphic eye-stalks found in many species. These flies are of additional interest because their X chromosome is derived largely from an autosomal arm in other flies. To identify candidate genes required for development of dimorphic eyestalks and investigate how sex-biased expression arose on the novel X, we compared gene expression between males and females using oligonucleotide microarrays and RNA from developing eyestalk tissue or adult heads in the dimorphic diopsid, *Teleopsis dalmanni.* Microarray analysis revealed sex-biased expression for 26% of 3,748 genes expressed in eye-antennal imaginal discs and concordant sex-biased expression for 86 genes in adult heads. Overall, 415 female-biased and 482 male-biased genes were associated with dimorphic eyestalk development but not differential expression in the adult head. Functional analysis revealed that male-biased genes are disproportionately associated with growth and mitochondrial function while female-biased genes are associated with cell differentiation and patterning or are novel transcripts. With regard to chromosomal effects, dosage compensation occurs by elevated expression of X-linked genes in males. Genes with female-biased expression were more common on the X and less common on autosomes than expected, while male-biased genes exhibited no chromosomal pattern. Rates of protein evolution were lower for female-biased genes but higher for genes that moved on or off the novel X chromosome. These findings cannot be due to meiotic sex chromosome inactivation or by constraints associated with dosage compensation. Instead, they could be consistent with sexual conflict in which female-biased genes on the novel X act primarily to reduce eyespan in females while other genes increase eyespan in both sexes. Additional information on sex-biased gene expression in other tissues and related sexually monomorphic species could confirm this interpretation.

## Introduction

The evolution of sexual dimorphism requires differential selection such that a trait advantageous in one sex is deleterious in the opposite sex [1]. When trait expression has a genetic basis, development of sexual dimorphism also requires differential gene expression between the sexes at some point during development. This can arise either by sex-biased expression or by genes relocating to a sex chromosome or both. How such sex-biased expression evolves depends on how gene expression influences traits and fitness in each sex and may be complex. Early population genetic models predicted that recessive alleles that benefit heterogametic males but are detrimental to homogametic females should increase when rare more easily on the X due to male hemizygosity [2], [3]. In addition, selection for female-beneficial alleles should be stronger for X-linked than autosomal genes because genes on the X chromosome are exposed to selection in males half as often as in females, assuming an equal sex ratio. These predictions change, however, if dominance varies [4] or is context dependent [5]. Furthermore, sexual selection is expected to operate on sex-linked loci differently than on autosomal loci [5], [6]. In particular, genes that affect expression of a sexually selected trait in males are generally expected to increase in frequency more rapidly when on an autosome or on the Y than on the X [6], although X chromosome segregation distortion can lead to the opposite prediction [7].

While agreement on the roles of the sex chromosomes underlying the evolution of sexual dimorphism has yet to be reached [8], consistent patterns of sex-biased expression have been reported. Multiple studies in *Drosophila* have reported that much of the genome exhibits sex-biased expression [9]–[12] with the amount depending on the method of analysis [13]. One of the most common findings is that the X chromosome contains fewer male-biased genes and more female-biased genes than expected based on chromosome content [13]–[16]. This pattern appears to have been driven primarily by gene duplication and chromosomal movement [16], although in *D. pseudoobscura* many ancestral autosomal male-biased genes have switched to become female-biased on the neo-X chromosomal arm [13]. Male-biased genes are also less common on the X than expected in mice [17], mosquitos [18] and flour beetles [19]. However, these patterns are not universal [20], [21] and sometimes differ by tissue [12]. For example, more male-biased genes are expressed in mice heads [11], [22], *Drosophila* heads [11], [22], and *Drosophila* accessory glands [13] than expected on the X.

Three hypotheses have been proposed to explain why the X may contain fewer male-biased and more female-biased genes than expected. First, sexually antagonistic selection should operate against the accumulation of male-beneficial genes on the X because selection on them is stronger in females. This hypothesis predicts that genes that move from the X to an autosome or Y should enhance male fitness and genes that move from an autosome to the X should enhance female fitness. Such gene movements can resolve sexual conflict via gene duplication and change in gene expression [23]. Microarray studies have revealed that the X in *Drosophila* contains genes with sexually antagonistic effects but sex-biased expression does not necessarily covary with sex-specific fitness and pleiotropy may limit the extent to which conflict can be resolved [24]. Second, dosage compensation may constrain the degree to which genes on the X can exhibit male-biased expression if gene dosage is achieved by hyper-expression of hemizygous males, as in *Drosophila* [25]. This hypothesis predicts that male-biased genes on the X are more likely the result of a reduction in female expression than an elevation of male expression, especially among highly expressed genes. Third, expression of X-linked genes during postmeiotic spermatogenesis may be inhibited due to transcriptional silencing [26]–[29]. This phenomenon, known as meiotic sex chromosome inactivation (MSCI), predicts that genes expressed in testes should relocate from the X to an autosome, a pattern that has been documented for flies [30], [31] and mammals [17], [32], [33].

These hypotheses are not mutually exclusive and their relative importance remains to be determined [12]. However, for several reasons MSCI is unlikely to provide a complete explanation for the biased chromosomal distribution of sex-biased genes. First, a reduction in male-biased genes on the X has been reported for genes that are expressed primarily in somatic tissue [10], [15], a pattern that should not be influenced by X-chromosome silencing in the germline. Second, excessive retrotransposition of testes-biased genes has also been found across different autosomes [34] indicating that chromosomal redistribution is not limited to movement off of the X. Finally, two recent studies in *Drosophila* have questioned the existence of MSCI [35], [36] suggesting instead that reduced levels of X-linked gene expression results from the absence of germline dosage compensation throughout spermatogenesis.

Further insight into the causes of sex-biased expression patterns requires information from additional species, especially those with extremely sexually dimorphic traits. Stalk-eyed flies in the family Diopsidae have become an iconic system for studying sexual selection and provide a particularly useful system for addressing this topic. Many species exhibit dramatic sexual dimorphism in head shape [37] with the outer-most distance between the eyes (eyespan) being two or more times the body length of males in some species. Sexual dimorphism in eyespan has evolved multiple times within the family [38] and is heritable [39], [40]. Sexual selection operates on male eyespan before mating in sexually dimorphic species by male-male competition [41] and female choice [42]–[44]. Sexual selection also operates after mating because both males and females are promiscuous [43], females store sperm from multiple males [45], [46], and male eyespan is associated with fertility [47], [48]. In addition, several species in the genus *Teleopsis* are polymorphic for X chromosome drive in which male carriers produce mostly female offspring [49], [50]. In *T. dalmanni* X drive is associated with reduced eyespan due to linkage [51] and as a consequence influences sexual selection [7].

Comparative genomic hybridization (CGH) [52] has revealed that the X in *Teleopsis* is derived from Muller element B (chromosome arm 2L in *D. melanogaster*). In addition, comparison of gene assignments to chromosomes in four species of *Teleopsis (T. dalmanni, T. quinqueguttata, T. thaii,* and *T. whitei)* indicates that some genes have moved onto or off of the X since these species diverged from a common ancestor with *Drosophila* approximately 70 MYA, and from each other [52]. Surprisingly, the majority of movement onto the X within *Teleopsis* is concentrated on the branches leading to the eyespan dimorphic taxa, while all of the movement off the X occurs in the eyespan monomorphic species. The presence of a novel X and gene movement between chromosomes raises questions about how gene expression is regulated between the sexes and the influence of gene movement on the expression of genes involved in head shape development. When an X chromosome arises, dosage compensation sometimes, but not always [53], evolves to equalize gene expression across the sexes. For example, *Drosophila* species with a neo-X, i.e. an ancestral X fused with an autosomal arm, studied to date utilize mechanisms already in place on the ancestral X rather than a new dosage compensation mechanism [54], [55].

The primary aim of this study is to identify candidate genes that are involved in the development of sexually dimorphic head shape and differ in expression between males and females. To this end we use custom-designed oligonucleotide microarrays to detect sex-biased gene expression in developing eyestalk tissue, i.e. the eye-antennal imaginal discs and brains [56] from wandering third-instar larvae of the dimorphic species, *T. dalmanni*. To identify sex-biased gene expression that is not occurring in all tissues and may be limited to the eye-antennal disc, we also assess gene expression in adult heads of *T. dalmanni*. Finally, we compare gene expression during eyestalk development to differential expression between lines selected for longer or shorter eyestalks [56] to determine if any of the genes that influence sexual dimorphism also influence heritable differences in eyestalk length among male *T. dalmanni*. These comparisons should, therefore, reveal candidate genes necessary for the development of elongated eyestalks in male *T. dalmanni*.

Given that these flies carry a novel X [52], a secondary aim is to determine how gene expression is influenced by chromosome location. By comparing levels of expression for X-linked and autosomal genes in each sex we determine if sex-biased expression depends on chromosome type and the extent to which dosage compensation exists. If dosage compensation is complete, then genes on the X should exhibit equal expression in males and females and expression of genes on the X should be equal to expression of genes on autosomes in males [53]. If dosage compensation is absent, then expression of X-linked genes in females should be greater (up to a factor of 2) than in males and expression of autosomal genes should be greater than X-linked genes in males. Because gene movement on and off the X has occurred [52], we also assess the relationship between gene movement and sex-biased expression. Finally, to assess the extent to which sex-biased genes may have been under selection, we compare rates of protein evolution between sex-biased and unbiased genes.

## Materials and Methods

### Fly stocks

Adult and larval flies were reared in the lab from large outbred populations of *Teleopsis dalmanni* (synonymized with Cyrtodiopsis, [57]) that were originally collected near the village of Ulu Gombak in peninsular Malaysia in 1999. The stock population was maintained at 25°C at 70% humidity with a 12 h L:D cycle and fed pureed corn twice weekly. Larvae were reared in cups containing 25–50 ml of pureed corn and kept in an incubator at 25°C with a 12 h L:D cycle. Eclosed flies were kept in small jars until at least 3 weeks of age.

### Ethics statement

Flies were cultured in the lab under a permit from the USDA Animal and Plant Health Inspection Service. These studies do not involve endangered or protected species.

### Sample preparation

Histological [58] and fate mapping studies [59] on diopsid flies indicate that eye-stalks develop from eye-antennal imaginal discs which undergo rapid growth at the end of the larval stage. In addition, application of synthetic juvenile hormone (methoprene) to pre-pupal flies caused a significant shift in male eyespan to body length allometry in adult flies [60], indicating that eye-stalk development can be altered by hormone titers in wandering larvae. Therefore, we collected imaginal disc and brain tissue from wandering larvae just after gut purge. To recognize larvae without food in their digestive tract we reared larvae on food that had been dyed with green food color. Using this technique, gut-purged larvae have transparent, instead of green, digestive tracts. Such larvae develop pupal cases in less than 8 h, so this selection process minimizes variation in gene expression caused by development.

Under a dissecting scope with fiber-optic illumination larvae were bisected in phosphate buffered saline (PBS) and the anterior end inverted to expose the brain and eye discs. We removed the pharyngeal jaws, eye-antennal discs, optic lobes, and brain as a complete unit and then stored the tissue in RNA*later*® (Applied Biosystems/Ambion, Austin, TX) in individually labeled tubes for a day prior to storage at −20°C. We also inverted the posterior end, removed the digestive tract and fatty tissue, and transferred the cuticle and remaining tissue into a tube containing 130 µl of distilled water before storage at 4°C. DNA was subsequently extracted from each sample using 10% Chelex [61].

To determine the sex of each larva, we used the polymerase chain reaction (PCR) to amplify an X-linked and a Y-linked marker. The presence or absence of the Y-linked product indicated either male or female, respectively, while the presence of the X-linked product confirmed successful amplification of DNA. The X-linked marker amplified a region associated with the gene *bunched* [c.f. 40] while the Y-linked marker involved a gene region identified from the CGH experiment [52] using the following primers (forward: 5′ -GATTCCAACATGCCCAATTC-3′, reverse: 5′-CACCGGAGAAACAGTTTGGT-3′) to amplify a 264 bp product in males. In a test run, these primers successfully amplified product in 47 male samples but failed to do so in 48 female samples; thus, this is a reliable method for sexing larvae. Each multiplexed 10 µl PCR reaction contained 1 µl of template DNA, 0.5 µM each primer (forward primers were fluorescently labeled), 1 X PCR buffer, 2.5 mM MgCl_2_, 0.2 mM dNTPs, 0.5 units of Taq, and distilled water up to 10 µl. To amplify DNA, we used a thermocycler (MJ Research PTC-100) with the following cycling conditions: 95°C for 2 min; 33 cycles of 94°C for 30 seconds, 58°C for 30 seconds, and 72°C for 30 seconds; and a final 72°C for 7 min. PCR products were separated on an ABI 3730xl (Applied Biosystems, Foster City, CA), and allele sizes were recorded using GeneMapper® v4.0 (Applied Biosystems, Foster City, CA, USA).

Each larval RNA sample contained eye-antennal imaginal disc plus brain tissue from 20–25 individuals of the same sex. Total RNA was extracted using the SV Total RNA Isolation System (Promega) and stored at −80°C. Each adult head RNA sample contained head capsules from six virgin flies of the same sex between 1 and 3 weeks of age. Heads were ground with a pestle under liquid nitrogen and RNA was extracted using the mirVANA total RNA extraction kit (Ambion).

The quality of each sample was checked with an Agilent 2100 Bioanalyzer. Only high-quality RNA samples (260/230 and 260/280>1.8 and RNA integrity above 7) were selected for hybridization. A total of ten pairs of larval samples and 12 pairs of adult head samples were hybridized, but only 11 head sample arrays were used in the analyses due to poor hybridization on one array resulting in low expression estimates.

### Microarray hybridization

#### Slide construction

The microarray platform used in this study was designed to measure the expression of 3,748 unique genes (see Table S1) involved in the development of *Teleopsis dalmanni* heads. Oligonucleotide probes 60 bp in length were selected from contig sequences obtained from an EST library made from the eye-antennal imaginal discs of larval and pupal stage *T. dalmanni* [56]. This study generated over 33,000 ESTs that assembled into 11,545 contigs, of which 3,491 had significant homology to a gene in *Drosophila*. Five 60 bp probes were designed for each of these genes. We also included probes to 149 contigs that had open reading frames greater than 450 bp (labeled ORF-# in Table S1) and 108 contigs that had relatively high representation in the EST database (>4 cDNA clones, labeled X-# in Table S1). As a result, each array had 18,203 unique probes each printed twice at random locations on an Agilent 4×44K format slide. Using CGH [52], we previously identified chromosome location for 3,417 of these genes with 2,891 being on an autosome, 525 on the X chromosome, 1 on the Y chromosome and 331 unknown. We used expression data from the autosomal and X-linked genes to assess sex-biased expression and dosage compensation as described below.

#### Hybridization

We used T7 to amplify messenger RNA prior to labeling [62]. This procedure uses reverse transcription to make single-stranded cDNA and then DNA polymerization to make double-stranded (ds) cDNA, which is then amplified using a linear amplification procedure. Each male or female sample was labeled alternately with Cy3 or Cy5 dye and then a pair of biologically independent male and female samples was hybridized to an oligoarray using an Agilent rotator rack and oven. Following Agilent's protocol, hybridizations were mixed with equal amounts of labeled nucleic acid. The ratio of the amount of labeled dye to nucleic acid (pmol/ug) was adjusted so that the same amount of red and green signal was acquired in each channel. To maximize signal detection the Agilent default PMT (photo-multiplier tube) setting of 100% for both red and green channels was used. After hybridization slides were scanned at 5 µm resolution using an Agilent G2539A scanner in an ozone-scrubbed room. RNA spike-in controls were mixed with each pair of samples and co-hybridized to each array following the manufacturer's instructions. The spike-in controls were two sets of ten synthesized RNA mixtures derived from the Adenovirus E1A transcriptome with different concentrations in each set.

#### Data analysis

Agilent's Feature Extraction Software was used for array image analysis and the calculation of spot intensity measurements. Features with saturated or high pixel variation were removed from further analysis. Processed signal intensities were calculated using a spatial detrend value for background subtraction and a combination of linear and lowess normalization to correct for any differences due to dye within an array. The percentage of features saturated (intensities greater than 65,502) in either channel for any of the arrays was less than 0.16%.

Processed signal intensity values were log_2_ transformed for each probe and then average intensity was calculated over probes for each gene. These methods produced replicable estimates of gene expression across tissues and species. To illustrate the consistency in expression estimates we calculated the average (± SD) correlation between log_2_ expression intensity across genes using different male or female samples. For the larval samples the cross array correlation was 0.964±0.027 (n = 190 pairs) and for the adult head samples it was 0.884±0.077(n = 231 pairs).

Sex-biased expression of genes was detected for each tissue comparison using two-class paired tests implemented with MeV v4.7 software [63] in which average male intensity was paired with average female intensity for each gene on an array. Significant sex-biased expression was assessed by permutation [64] such that either the False Discovery Rate (FDR) did not exceed 5% or the median number of genes estimated to be false did not exceed 5 [65]. The data in this publication are available at NCBI's Gene Expression Omnibus, accession number GSE37121 (http://www.ncbi.nlm.nih.gov/geo/query/acc.cgi?acc=GSE37121).

Functional analysis of over-represented Gene Ontology (GO) categories was conducted using GeneMerge [66]. Only GO categories that had a relatively unique set of sex-biased genes (greater than 25% of their genes) relative to all of the genes present in more significant categories are presented.

To determine if sex-biased gene expression is related to change in male eyespan we compared the direction (male, female or none) of sex-biased expression in *T. dalmanni* eye discs to differential expression between male flies selected for long or short eyespan over 60 generations from a previous study [56] using a contingency table test. In both studies gene expression was measured from the same source tissue, i.e. eye-antennal discs dissected from wandering larvae, using custom-designed Agilent 44K microarrays containing probes designed to assay many of the same genes. The two array platforms had 3,099 genes in common.

To determine if the distribution of sex-biased genes was independent of chromosome type we used contingency table tests to compare the proportion of genes that were either autosomal or X-linked, as determined by CGH chromosome assignments [52] for each tissue.

Gene movement was inferred to have occurred since the origin of the novel X in an ancestor to *T. dalmanni* by reference to gene location in *Drosophila melanogaster* and *Anopheles gambiae*. Because 90% of the annotated X-linked genes in *T. dalmanni* are found on chromosome arm 2L in *D. melanogaster* and 3R in *A. gambiae* [18], [52], we assume that genes on chromosome arm 2L in *D. melanogaster* were on an ancestral X chromosome and must have moved if they are now located on a *T. dalmanni* autosome. *De novo* assembly of RNA sequences from Illumina sequencing data obtained from testes and adult heads revealed that many gene movements involve a duplicate gene copy [67] as would be expected if movement is caused by retrotransposition. We then compared the pattern of sex-bias for genes inferred to have moved onto or off the novel X using contingency table tests and either the distribution of genes on the autosomes or on the X as the expectation.

The effects of gene movement and sex-bias on the relative rate of protein evolution were determined using ANOVA. The relative rate of protein evolution in the lineage leading to *T. dalmanni* for 2,599 genes on the array was estimated from maximum likelihood trees constructed from amino acid data (using a JTT substitution model with no invariant sites) for *A. gambiae*, three *Drosophila* species – *D. melanogaster, D. pseudoobscura* and *D. virilis* – and *T. dalmanni.* The index of protein evolution was calculated by dividing the length of the branch leading to *T. dalmanni* by the length of the entire tree [56].

We used JMP v5.0.1.2 [68] for all other statistical analyses.

## Results

### Sex-biased expression in larval and adult heads

**Figure 1:**
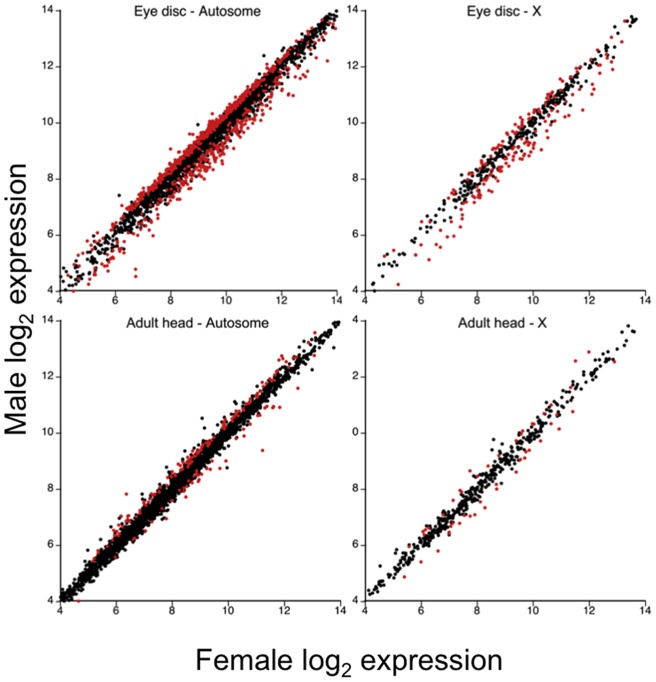
Male versus female log_2_ expression for autosomal and X-linked genes by tissue. Genes exhibiting significant sex-biased expression with FDR <0.05% are shown in red.

Examination of hybridization patterns for eye-antennal imaginal disc and adult head tissue samples indicated that most of the 3,748 genes on the array were expressed in both tissues (cf. Figure 1). The percent of genes that exhibited normalized log_2_ expression greater than five was 98.2% for eye discs and 94.7% for adult heads.

Analysis of average log_2_ gene expression intensity between the sexes revealed genes with sex-biased expression in larval and adult head tissue (Figure 1). Matched pairs comparisons for eye disc tissue revealed 985 genes that exhibited sex-biased expression with 446 female-biased and 539 male-biased (see Table S1 for gene names). In contrast, only 242 genes on the array exhibited sex-biased expression in adult heads. Contingency table analysis indicated that sex-biased expression exhibited significant concordance among genes across tissues (Table 1, *X^2^* = 124.9, d.f.  = 4, P<0.0001). A total of 86 genes displayed the same sex-biased expression pattern in both adult and larval tissue while only two genes showed a reversal in sex-biased expression. The two genes were CG10359 and CG13335, both of which were male-biased in larvae and female-biased in adults. Thus, taking the results from both tissue comparisons together, 897 genes showed sex-biased expression in developing eyestalks but not in adult heads.

**Table 1:** Observed (and expected) number of sex-biased genes in larval and adult heads.

	Larval eye discs			
Adult heads	Female-bias	No bias	Male-bias	Total
Female-bias	31(9.3)	45(57.5)	2(11.2)	78
No bias	415(417.2)	2609(2584.6)	482(504.2)	3506
Male-bias	0(19.5)	109(120.9)	55(23.6)	164
Total	446	2763	539	3748

Among the sex-biased genes in larval heads, a total of 817 could be annotated, based on Blast hits, as homologous to a protein in *D. melanogaster*. Functional analysis of these genes indicated that, at this late larval stage, female eye disc development is distinguished more by differentiation and patterning while male eye disc development is focused more on growth (Table 2). Female-biased genes were significantly overrepresented for genes involved in transcription, anatomical development and cell communication, while male-biased genes were overrepresented for genes involved in metabolism. Consistent with these biological process categories, there was a distinct difference between the sexes in the predicted cellular location of sex-biased gene expression. Gene ontology information indicated that female-biased expression is concentrated in the nucleus with 91 sex-biased genes falling into this category compared to only a single male-biased gene. In contrast, genes associated with mitochondrial function tend to exhibit male-bias with 51 genes in this category while none of the female-biased genes are known to influence the function of this organelle.

**Table 2:** Over-represented gene ontology (GO) categories among sex-biased genes*.

GO term	Description	Sex-bias	#	Array freq	Bias freq	E-value
Biological Processes:						
GO:0006350	transcription	Female	73	0.103	0.202	2.29E–06
GO:0048856	anatomical structure development	Female	88	0.149	0.244	3.78E–04
GO:0007154	cell communication	Female	87	0.151	0.241	0.0015
GO:0019752	carboxylic acid metabolic process	Male	37	0.035	0.070	0.0157
GO:0051186	cofactor metabolic process	Male	26	0.022	0.049	0.0305
GO:0006629	lipid metabolic process	Male	33	0.031	0.062	0.0476
Cellular Component:						
GO:0005634	nucleus	Female	91	0.159	0.252	0.0001
GO:0005739	mitochondrion	Male	51	0.051	0.096	0.0006

# – number of sex-biased genes in that category.

Array freq – proportion of annotated genes on the array that fall into that category.

Bias freq – proportion of genes that are sex-biased that fall into that category.

* See Table S2 for gene names included in each category.

Of the remaining 80 genes that were sex-biased only in larval heads and could not be matched to any *D. melanogaster* protein, 76 were female-biased and only four were male-biased, a pattern that differed greatly from the annotated genes in which male-biased genes were more common (*X^2^* = 111.2, d.f.  = 1, P<0.0001). Unknown genes also exhibited more sex-biased expression (31.9%) than annotated genes (24.0%, *X^2^*  = 7.90, d.f.  = 1, P = 0.005). Thus, genes apparently unique to diopsids were more likely to be sex-biased and exhibit female-biased expression in the developing head.

The direction of sex-biased expression in developing eyestalks was strongly related to differential expression between male flies from lines selected for long or short eyespan (Table 3, *X^2^* = 33.9, d.f.  = 4, P<0.0001). In particular, genes that exhibited elevated expression in flies selected for short eyestalks were female-biased more often than expected (*X^2^* = 20.0, d.f.  = 1, P<0.0001) and male-biased less often than expected (*X^2^* = 7.5, d.f.  = 1, P<0.0001).

**Table 3:** Observed (and expected) number of genes exhibiting sex-bias and differential expression between selected lines.

Selected-line†	Female-bias	No bias	Male-bias	Total
Short eye-stalks (log_2_ H/L <0)	69(40.5)***	247(255.7)	32(51.8)***	348
None (log_2_ H/L = 0)	262(279.0)	1766(1759.7)	367(356.2)	2395
Long eye-stalks (log_2_ H/L >0)	30(41.4)	264(261.6)	62(52.6)	356
Total	361	2277	461	3748

† refers to lines of flies selected for long (H) or short (L) male eyestalks for 60 generations [56]. *** P<0.0001 for cell chi-square value.

### Dosage compensation

Comparison of female to male log_2_ gene expression on the X using matched pairs tests revealed that male expression is slightly, but significantly, lower than female expression for X-linked genes in larval tissue (t_524_ = 3.56, P<0.0004; mean (± SE) male expression  = 9.30±0.01, mean female expression  = 9.36±0.01) but not adult head tissue (t_524_ = −0.70, P = 0.485; mean male expression  = 8.23±0.01, mean female expression  = 8.22±0.01). Comparison of log_2_ expression between X and autosomal genes in males revealed no difference for larval tissue (t_3414_ = 1.59, P = 0.113; mean autosomal expression  = 9.48±0.04, mean X expression  = 9.30±0.10) or adult heads (t_3414_ = 0.15, P = 0.88; mean autosomal expression  = 8.21±0.04, mean X expression  = 8.23±0.10). Thus, these results are largely consistent with widespread dosage compensation. As in *Drosophila*, the mechanism for dosage compensation appears to involve hyper-transcription of the X given that the average expression in males of X-linked genes did not differ from the average expression of autosomal genes.

**Figure 2:**
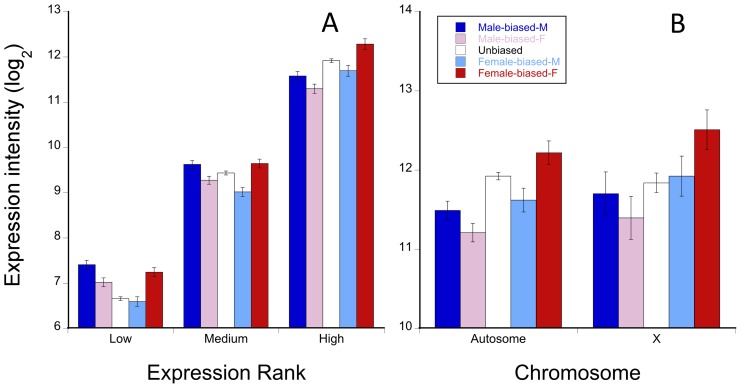
Average log_2_ gene expression by sex, sex-bias and expression rank. A) Mean male (M) and female (F) log_2_ expression intensity (± SE) in larvae for sex-biased and unbiased genes subdivided into thirds by expression rank. B) High expression rank data shown in (A) divided by chromosome type.

To determine if the mechanism for dosage compensation in males might constrain sex-biased expression in *T. dalmanni* we compared average log_2_ expression intensity for males and females using direction of sex-bias in eye discs, type of chromosome, and expression rank (in which the list of genes was ranked by average expression and categorized into thirds) as factors in two analyses of variance. The results were similar for expression in males or females. Sex-bias direction (males: F_2,3398_ = 8.92, P = 0.0002; females: F_2,3398_ = 17.36, P<0.0001), expression rank (males: F_2,3398_ = 1308, P<0.0001; females: F_2,3398_ = 1305, P<0.0001), and their interaction (males: F_4,3398_ = 6.22, P<0.0001; females: F_4,3398_ = 5.26, P = 0.0003) were highly significant. But, neither chromosome type nor any interaction involving chromosome type was significant for either male or female expression. Relative to unbiased genes, male-biased genes showed elevated expression in males when they have low or medium but not high expression intensity while female-biased genes exhibited elevated expression in females relative to unbiased genes at all levels of expression (Figure 2A). However, among highly expressed genes, expression of male-biased genes on the X did not differ from expression of male-biased genes on the autosomes (Figure 2B). These results are inconsistent with the hypothesis that male-biased expression is constrained on the X because dosage compensation in males is due to hyper-transcription [25]. Levels of expression of sex-biased genes did vary by sex but not as a consequence of type of chromosome.

### Chromosome location, movement, and sex-biased expression

**Figure 3:**
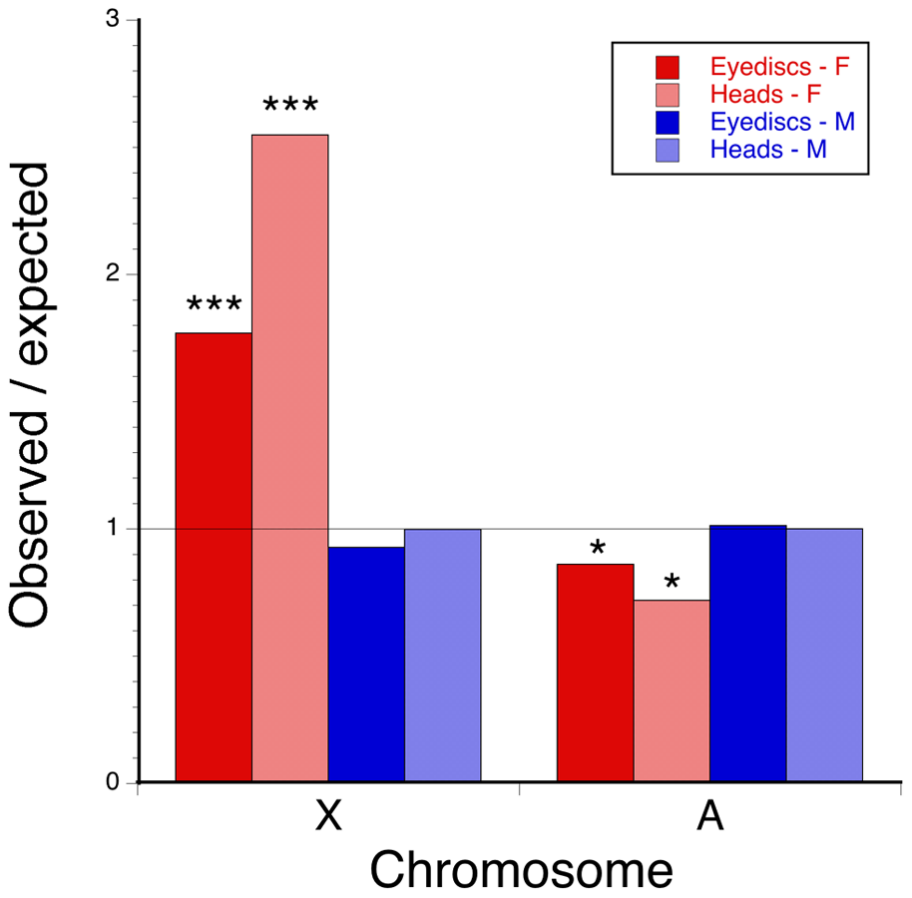
Observed/expected number of sex-biased genes by chromosome for eye disc or adult head tissue. F  =  female-biased, M  =  male-biased and line indicates no effect. * P<0.05, *** P<0.001.

The relative frequency of sex-biased genes differed between the autosomes and the X chromosome consistently across tissues. In larval tissues a contingency table test indicated that the proportion of sex-biased genes differed depending on type of chromosome (*X^2^* = 46.4, d.f.  = 2, P<0.0001). Female-biased genes were more common on the X and less common on the autosomes than expected, whereas male-biased genes occurred on both types of chromosomes in proportion to chromosome gene content (Figure 3). Similarly, sex-biased genes were associated with chromosome type in adult heads (*X^2^* = 29.6, d.f.  = 2, P<0.0001) with female-biased genes more common on the X and less common on the autosomes than expected, and male-biased genes present in proportion to chromosome content. Cross-classifying genes by the type of sex-biased expression in each tissue and comparing the resulting eight sex-biased categories against chromosome revealed a highly significant association (*X^2^* = 80.9, d.f.  = 7, P<0.0001). This result was due largely to genes with female-biased expression in both tissues being over-represented on the X (observed 17, expected 4.3, cell *X^2^* = 37.5, d.f.  = 1, P<0.0001) but under-represented on the autosomes (observed 11, expected 23.7, cell *X^2^* = 6.8, d.f.  = 1, P<0.01) and genes that were female-biased in larvae but unbiased in adults being over-represented on the X (observed 89, expected 55.9, cell *X^2^* = 19.5, d.f.  = 1, P<0.001). Genes with male-biased expression in one or both tissues occurred on each type of chromosome in proportion to their abundance.

To determine how gene movement influences gene expression we compared the proportions of sex-biased and unbiased genes that have either moved onto or off of the X relative to the sex-biased and unbiased proportions on the *T. dalmanni* X or autosomes using contingency table analyses. For these tests we scored a gene as male- or female-biased if it was differentially expressed in either tissue examined in this study. The two genes that had contrasting sex-biased expression patterns between tissues were excluded. The contingency table for autosomal genes was significant (*X^2^* = 6.6, d.f.  = 2, P = 0.036). Genes inferred to be on the ancestral X, i.e. chromosome arm 2L in *D. melanogaster*, but now autosomal in *T. dalmanni* (labeled as Onto A, Table 4) were less likely to be female-biased and more likely to be male-biased than expected when compared to other autosomal genes. In contrast, the contingency table for X-linked genes was not significant (*X^2^* = 3.3, d.f.  = 2, P = 0.189) indicating that genes that have moved from an ancestral autosome, i.e. not on chromosome arm 2L in *D. melanogaster* (labeled as Onto X, Table 4), now exhibit the same pattern of sex-biased expression as other X-linked genes. The distribution of sex-biased genes among the 135 genes that either moved onto or off of the X did not differ from the pattern exhibited by genes that have not moved (*X^2^* = 1.67, d.f.  = 2, P = 0.433).

**Table 4:** Observed and expected& number of annotated genes by sex-bias and inferred chromosome history.

		Chromosome history#			
Sex-bias†	A	Onto A	Onto X	X	Total
Male-bias	493 (500)	23 (16.1)	6 [7.9]	74 [72.1]	596
No bias	1905 (1904)	60 (61.5)	28 [30.9]	284 [281.1]	2277
Female-bias	266 (260.6)	3 (8.4)	15 [10.2]	88 [92.8]	372
Total	2664	86	49	446	3245

& Expected numbers are derived from contingency tables using either (autosomal) or [X-linked] gene totals.

† Sex-bias refers to expression in either eye disc or adult head tissue.

# Onto X and Onto A indicate genes inferred to have moved onto or off of the novel X based on synteny with chromosome arm 2L in *D. melanogaster.*

**Figure 4:**
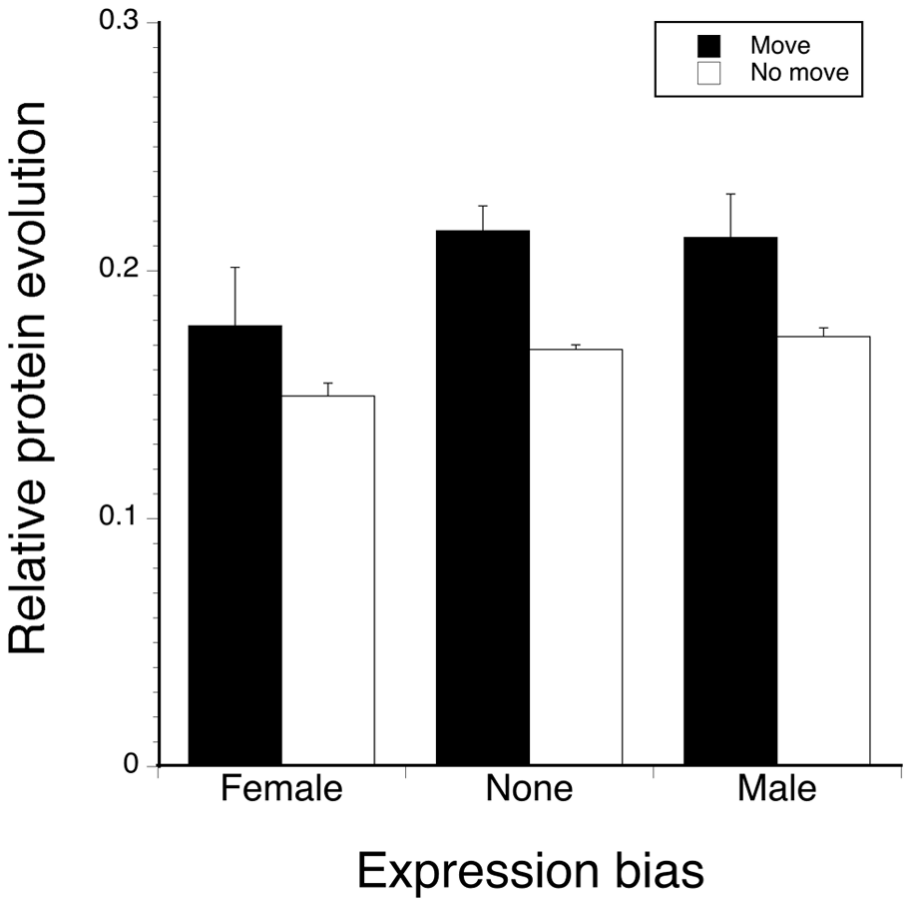
Rate of protein evolution for sex-biased genes by chromosome history. The average rate of protein evolution (± SE) was measured as the branch length leading to *T. dalmanni* expressed as a percent of the tree length obtained from a phylogenetic analysis including *A. gambiae* and three *Drosophila* species (cf. [52]).

Sex-biased expression and chromosome movement were both associated with evolutionary change in protein sequences for annotated genes. A two-way analysis of variance on relative branch lengths using sex-bias direction and chromosome movement was highly significant (F_5, 2466_ = 11.94, P<0.0001) with both gene movement (F_3, 2484_ = 14.83, P<0.0001) and sex bias (F_2, 2484_ = 7.94, P = 0.0004) but not their interaction explaining significant amounts of variation. Genes that have moved between a sex chromosome and autosome exhibited a higher rate of protein evolution (Tukey HSD posthoc test, Figure 4). In addition, genes that showed female-biased expression in either tissue were evolving more slowly, i.e. have proportionally shorter branch lengths leading to *T. dalmanni* (Tukey HSD posthoc test). Genes with male-biased expression were evolving faster but did not differ significantly from genes that did not exhibit any sex bias in expression. Slower protein evolution for genes with female-biased expression also occurs in *Drosophila* [69].

## Discussion

The microarray results presented here provide a comprehensive catalogue of gene expression differences between the sexes in the larval tissue that develops into sexually dimorphic eyestalks. A considerable fraction (26%) of the genes examined exhibit sex-biased expression, with most of this differential expression specific to the eye-antennal discs as compared to the adult head. Notably, though, the magnitude of sex-bias is modest, with few genes exhibiting more than a two-fold difference in expression (cf. Figure 1). Despite widespread examination of sex-biased gene expression in *Drosophila* (e.g. [9]–[13]) few studies have compared male and female gene expression in developing somatic tissue for a trait that is sexually dimorphic in adults. In one study, Barmina et al. [70] examined gene expression in the pupal imaginal disc of the first leg, which contains sexually dimorphic bristle patterns, and the second leg, which is not sexually dimorphic. They found moderate levels of sex-biased gene expression (∼100 genes) in the dimorphic tissue and no sex-biased expression in the monomorphic leg [70]. Another study that focused on gene expression in several somatic larval tissues, found low levels of sex-biased gene expression associated with metamorphosis [71]. Thus, the results presented here suggest that the amount of sex-biased gene expression in the developing heads of *T. dalmanni* is equal to or greater than that measured at similar stages in *Drosophila*.

The abundant sex-biased expression in the eye-antennal discs of *T. dalmanni* is consistent with the late larval stage being a critical developmental period influencing morphological differences between the adult sexes. This inference is supported by the functional analysis of differential gene expression, which shows that expression in females is enriched for genes involved in patterning and differentiation while expression in males is dominated by genes involved in growth. Given the larger eyestalks in *T. dalmanni* males, increased expression of genes associated with growth in this tissue is not surprising. Eyestalks are of central importance to the breeding system in stalk-eyed flies as they influence a male's ability to gain access to females and may provide a signal that females can use to assess male quality [72]–[74]. In addition to eyestalk sexual dimorphism, substantial phenotypic and genetic variation exists for male eyespan. In this study, we found that the direction of sex-biased expression among genes is positively related to differential expression between male flies from lines selected for long or short eyespan [56] indicating that at least some of the genes responsible for sexual dimorphism also influence intraspecific variation in male eyestalk length.

As has been frequently found in *Drosophila* [11]–[13], [15], [16], X-linked genes show evidence of feminization in *T. dalmanni.* The stalk-eyed fly data differ, though, in that male-biased genes occur in proportion to chromosome content rather than below expectation on the X as in *Drosophila* [12], [15], [16]. Furthermore, because of the shared history between the *Teleopsis* X and *D. melanogaster* 2L, which has higher than expected numbers of male-biased genes in larval tissues [12], feminization of *T. dalmanni* X-linked genes must have occurred largely by a change in gene expression rather than by gene movement. The pattern of sex-biased expression among genes that have moved chromosomes is consistent with these chromosomal effects, i.e. female-biased expression is more common among genes that moved onto the X and less common among genes that have moved onto an autosome (cf. Table 4). Thus, either sex-biased expression has changed since movements occurred or sex-biased genes have moved preferentially. Gene expression measurements from additional species in which genes have moved more recently could reveal which of these possibilities occurs more often.

These results cannot be explained by meiotic sex chromosome inactivation (MSCI) because gametic tissues were not involved. Furthermore, expression of male-biased genes does not differ by type of chromosome – even among highly expressed genes – so constraints associated with dosage compensation [25] are also unlikely to explain why female-biased genes are more common on the X. Given that most sex-biased gene expression in eye-antennal imaginal discs appears to be related to the development of a sexually dimorphic trait, sexual conflict resolution remains as a potential explanation for the distribution of sex-biased genes.

Sexual conflict will occur if genes with beneficial effects in males have deleterious effects in females or vice versa. In an influential paper, Rice [3] pointed out that sexual dimorphism in a trait, such as eyespan, can evolve from a sexually monomorphic state either by males, but not females, developing long eyespan (pleiotropy mechanism) or by long eyespan first developing in both sexes followed by a decrease in female eyespan as a consequence of modifiers that limit expression to males (2-step modifier mechanism). In order to relate these population genetic predictions to measures of gene expression, sex-biased gene expression needs to influence the phenotypic expression of a dimorphic trait. The extent to which this assumption is true is unknown for most species. However, in stalk-eyed flies, we know that the genes measured in this study influence head development in general, and at least for some genes, the length of male eyestalks in particular (cf. Table 3). Because males with longer eyestalks are more successful at mating [43], [44], we can infer that expression of genes that increase eyespan will be favored in males. To the extent that long eyespan is also deleterious, perhaps through its effects on flight performance and predator avoidance [75], [76], it is plausible that expression of these genes could have deleterious phenotypic consequences in females. If sexually dimorphic eyestalks evolved via Rice's modifier mechanism, then expression of genes that decrease female eyespan, which would have beneficial phenotypic effects for females but not males, should be particularly abundant on the X. While Rice's 2-step model fails to consider context-dependent dominance or recombination [5], [6], this hypothetical scenario predicts an abundance of female-biased genes on the X chromosome such as we found.

Sexual conflict at genes with pleiotropic effects could be difficult to resolve [77], [78]. This study examined gene expression in only two tissues, so we have limited ability to determine if pleiotropic effects constrain change in gene expression in this species. Nevertheless, we were surprised that only two genes exhibited evidence of antagonistic expression between larval and adult head tissue whereas 86(of 985) genes exhibited concordant sex-biased expression in both tissues. Analysis of the transcriptome from a wider array of tissues across development would help reveal the extent to which additional genes that affect eyestalks exhibit pleiotropic effects. Gene duplication, in particular, may provide a powerful mechanism for resolving sexual conflict [23] and a recent analysis of RNA-seq data in *T. dalmanni* revealed that genes that have duplicated in stalk-eyed flies are more likely to exhibit sex-biased gene expression [67].

In addition to finding that genes with female-biased expression are more abundant on the X chromosome, genetic mapping studies have shown that the X chromosome and one of the autosomes carry genes with major effects on the length of male eyespan [51], [79], [80]. These results could be consistent with the modifier mechanism for the evolution of sexual dimorphism described above if autosomal genes cause an increase in eyespan in both sexes while X-linked genes act primarily to reduce eyespan in females. Whether some X-linked genes also are responsible for increases in male eyespan is less clear, although this could occur if X-linked transcription factors influenced expression of autosomal genes. Considerable allelic variation in transcription factors that contain amino-acid repeats has been detected and at least one X-linked variant has been associated with eyestalk length in *T. dalmanni* [40]. Additional studies on how allelic variation at candidate genes influences sex-biased expression are likely to provide insight into the nature of genetic change required to produce the extraordinary morphology displayed by these flies.

## Supporting Information

Table S1 **Summary of sex-biased expression values by gene.** Note: average normalized log_2_ intensity values, log_2_ ratios of male to female expression intensity, and type of sex-biased expression for eye discs and adult heads listed by gene name and chromosome location (X vs A) as inferred by CGH. Flybase ID (http://flybase.org/) refers to the gene identification number for the corresponding *Drosophila melanogaster* gene.
(XLS)

Table S2 **Gene names associated with significant gene ontologies.** Note: annotation symbol and gene name associated with each over-represented gene ontology category listed in Table 2. Note: biological process genes and cellular compartment genes are listed on separate sheets.
(XLS)
